# Association between the oxidative stress gene polymorphism and chronic obstructive pulmonary disease risk: a meta-analysis

**DOI:** 10.1186/s12890-023-02625-y

**Published:** 2023-10-10

**Authors:** Ting Zhou, Qiunan Zuo, Mengchun Chen, Yingying Zhao, Xiaohui Li, Shujin Guo

**Affiliations:** 1 Department of Geriatric Respiratory, School of Medicine, Sichuan Provincial People’s Hospital, University of Electronic Science and Technology of China, Chengdu, 611731 China; 2 Chengdu University of Traditional Chinese Medicine, Chengdu, 610075 China; 3 Department of Health Management & Institute of Health Management, Sichuan Provincial People’s Hospital, School of Medicine, University of Electronic Science and Technology of China, Chengdu, 611731 China

**Keywords:** COPD, Oxidative stress, Polymorphism, Risk, Meta-analysis

## Abstract

**Background:**

The association between the oxidative stress gene polymorphism and chronic obstructive pulmonary disease (COPD) risk has been extensively studied but the results have been controversial. This study aimed to investigate the overall association between the oxidative stress gene including glutathione S-transferase (GST), epoxide hydrolase exon (EPHX), superoxide dismutase (SOD), catalase (CAT), cytochrome P450 system (CYP) and heme oxygenase (HO-1) polymorphism and the risk of COPD.

**Methods:**

We searched the PubMed and EMBASE database to identify studies that investigated the association between the oxidative stress gene polymorphism and risk of COPD. The relevant data were extracted and statistical analyses were performed using the Revman 5.4 and STATA 12 software. Dominant genetic model, recessive model, co-dominant model, heterozygote model, and allele model were analyzed. Venice criteria and publication bias were conducted to access the credibility and reliability.

**Results:**

In total, 63 publications including 14,733 patients and 50,570 controls were included in the meta-analysis.15 genetic variants of 6 genes were analyzed, and 7 SNPs in GSTP1, CAT, CYP, SOD were first analyses until now. In our study, EPHX T113C C allele, GSTM1 null, GSTT1 null, GSTP1 A313G G and C341T T allele, CYP1A1 MspI C allele, SOD3 A213G G allele and L type in Ho-1 showed increased COPD risk, especially in Asians. T allele in CAT C262T and C allele in SOD2 Val 9 Ala were associated with decreased COPD risk. To avoid high heterogeneity and publications bias, subgroups analysis was performed in accord with HWE and ethnicity. Publication bias was assessed by Begg’s funnel plots and Egger’s test, and no publication bias were found for recessive models. 4 variants were identified with strong levels of epidemiological evidence of associations with the COPD risk.

**Conclusions:**

Our results confirm that oxidative stress gene polymorphism was associated with COPD risk. These finding can improve human understanding of this disease gene molecular level and enable early intervention and prevention of COPD. Well-designed studies with large sample sizes are essential to clarify the association of these significant variants with the susceptibility to COPD.

**Supplementary Information:**

The online version contains supplementary material available at 10.1186/s12890-023-02625-y.

## Introduction

Chronic obstructive pulmonary disease (COPD) is a progressing chronic respiratory disease which has become the third leading cause of death worldwide [1]. The prevalence of COPD in China was 13.6% in 2015, and the incidence is still increasing [2]. Etiologies and pathogenesis are important for COPD prevention and treatment. Environment factors, host genetics, oxidative stress, inflammation response, autoimmunity are indicated as risk factor for COPD development [3]. Smoking is a key factor for COPD pathogenesis, it can bring a lot of oxygen free radicals into the lungs, triggering oxidative stress and directly damaging lung tissue; while in the antioxidant system, antioxidant enzymes (including: glutathione S-transferase (GST), epoxide hydrolase exon (EPHX), superoxide dismutase (SOD), catalase (CAT) and cytochrome P450 system (CYP), etc.) provide endogenous biological defense against oxidative stress [4]. An imbalance between the oxidants and antioxidants leads to increased expression of genes involved in inflammation, increased secretion of airway mucus, and inactivation of anti-proteases [5]. Recently, a report recommends that COPD should be classified into five different types based on the main risk factors (genetics, early life events, lung infections, tobacco exposure and air pollution), for early intervention and prevention [6]. Genome-wide association study (GWAS) could identify sequence variations known as SNP (Single-nucleotide polymorphism) across the human genome [7]. It had been found that single nucleotide polymorphisms (SNPs) of oxidative stress enzyme genes may modify the ability to resist oxidative damage which may affect the COPD susceptibility [8].

Several susceptibility genes associated with oxidative stress have been investigated in genetic association case–control studies or prospective longitudinal cohort studies, however, the results have been inconclusive partly because of limitation of population size, ethnicities, and other confounding factors [9–12]. GWAS and meta-analyses are reliable and credible methos for the exploring of the pathogenesis of respiratory disease, and contribute to the accurate prevention and treatment for these diseases. Despite the existence of limitations in meta-analyses such as heterogeneity and publication bias, this method can provide more reliable results for gene polymorphisms study. Meta-analyses can improve statistical power and avoid error sources by summarizing related studies. Several published meta-analyses had reported the association between oxidative stress related genes with COPD susceptibility [9–12]. However, no systematic review investigating all oxidative stress related variants has been published until now.

In this study, we performed this latest and most comprehensive meta-analysis with accurate data and all eligible case–control studies to provide evidence-based information on the pathogenesis of COPD for potential strategies for its diagnosis, prevention and treatment. This study provides a more reliable and comprehensive conclusion about the association between the oxidative stress related gene polymorphism and COPD risk.

## Methods

Since this study was a meta-analysis based on published articles, we did not draft a statement of patient consent or seek the approval of internal review boards. The study was conducted according to the Preferred Reporting Items for Systematic Reviews and Meta-analyses (PRISMA) and was registered with PROSPERO (ID: CRD42023453464).

### Study identification and selection

A comprehensive literature search was conducted to identify all eligible studies that investigated the association between the enzymatic antioxidants gene polymorphism and COPD risk in PubMed and EMBASE database updated on April 27, 2023. The search terms used were as follows: “chronic obstructive pulmonary disease or COPD” in combination with “polymorphism or variant or mutation” and “oxidative or anti-oxidative or GST or EPHX or SOD or CAT or CYP or Heme Oxygenase-1 (HO-1)”. Reference lists of relevant were also manual checked for potential eligible studies.

### Inclusion and exclusion criteria

The inclusion criteria were as follows: (1) they were case–control genetic studies, (2) they evaluated the association between the enzymatic antioxidant genes polymorphism and COPD risk, and (3) the genotype distributions for cases and controls were sufficient to estimate the odd’s ratio (OR) with a 95% confidence interval (95%CI), and (4) these studies had only been conducted in human beings. The exclusion criteria were as follows: (1) abstracts, review articles, and letters, (2) genotype frequency not shown, and (3) repeated data. And (4) missing data that could be extracted from texts, tables, or chats, or requested by contacting the authors, otherwise these studies were excluded.

### Data extraction

To reduce bias and improve accuracy, two independent authors (Ting Zhou and Shujin Guo) checked all the included studies based on our inclusion and exclusion criteria. Any disagreements were solved by discussing and reached a consensus on every item finally. A data extraction table was made and the following data were extracted from the included studies: the name of the first author, year of publication, country of origin, ethnicity, gene assay method, sample size of case group and control group, total number and distribution of genotypes.

### Literature quality assessment

The Newcastle–Ottawa scale (NOS) evaluates the quality of the literature of case–control studies and cohort studies by means of 8 entries in three dimensions, including 4 entries for study population selection, 1 entry for comparability between groups, and 3 entries for exposure or outcome evaluation, with a maximum of 2 stars except for one entry for comparability between groups, and a maximum of 1 star for all other entries. The quality of the literature was evaluated using the semi-quantitative principle of the star system, with a full score of 9 stars. Two independent researchers judged whether the specific content of the included literature complied with the entries based on the NOS scale, and the more entries complied, the higher the total number of stars and the higher the quality of the literature.

### Statistical analysis

Methodologies for the meta-analysis were conducted as our previous article [13]. The strength of the association between the enzymatic antioxidants gene polymorphism and COPD risk was assessed by the odds ratio (OR) with its corresponding 95% confidence interval (95%CI). Random-effects (DerSimonian and Laird method) or fixed-effects model (Mantel–Haenszel method) was applied to pool the OR values according to the results of the heterogeneity examination. Heterogeneity was evaluated by an *χ*^*2*^-based Q statistic and I^2^, and a *p* value < 0.10 was statistically significant. Random-effects model was calculated when the pooled OR for *p* < 0.10. Otherwise, a fixed-effects model was used. The degree of heterogeneity was estimated with the I^2^ statistic and a value > 50% was considered as a large degree of heterogeneity. The significance of the pooled OR was evaluated by a Z-test with a *p* < 0.05. The dominant genetic model (D), recessive model (R), co-dominant model (CD), heterozygote model (H), and allele model (A) were used to assess the association of each genotype with the risk of COPD.

Pearson’s *χ*^*2*^ test was used to calculate the Hardy–Weinberg equilibrium (HWE). A *p* value of < 0.05 indicated deviation from HWE.

To ameliorate the effect of high heterogeneity, we conducted subgroup analysis. Subgroup analyses were performed for accordance with HWE and in different ethnic groups. In subgroup accordance with HWE, the *p* value was calculated in every variant, withing *p* value > 0.05 was included. In subgroups with different ethnicity, we extracted the ethnic information from the articles, and divided the populations to different groups according to the Caucasian, Asian, Latin and Africa.

Begg’s funnel plots and Egger’s test was tested for assessing the Publication bias. The reliability of a meta-analysis was evaluated by sensitivity analyses. Revman 5.4 software (Review Manager, Version 5.4, the Nordic Cochrane Centre, the Cochrane Collaboration, Copenhagen, 2008) and the STATA 12.0 software (Statistical Software, Release 12.0, College Station, TX: StataCorp LP, American, 2009) was applied for the meta-analysis. The Venice criteria were used to assess the level of strength to evaluate the association between the epidemiological and COPD risk [14].

## Results

### Characteristics of included studies

**Fig. 1:**
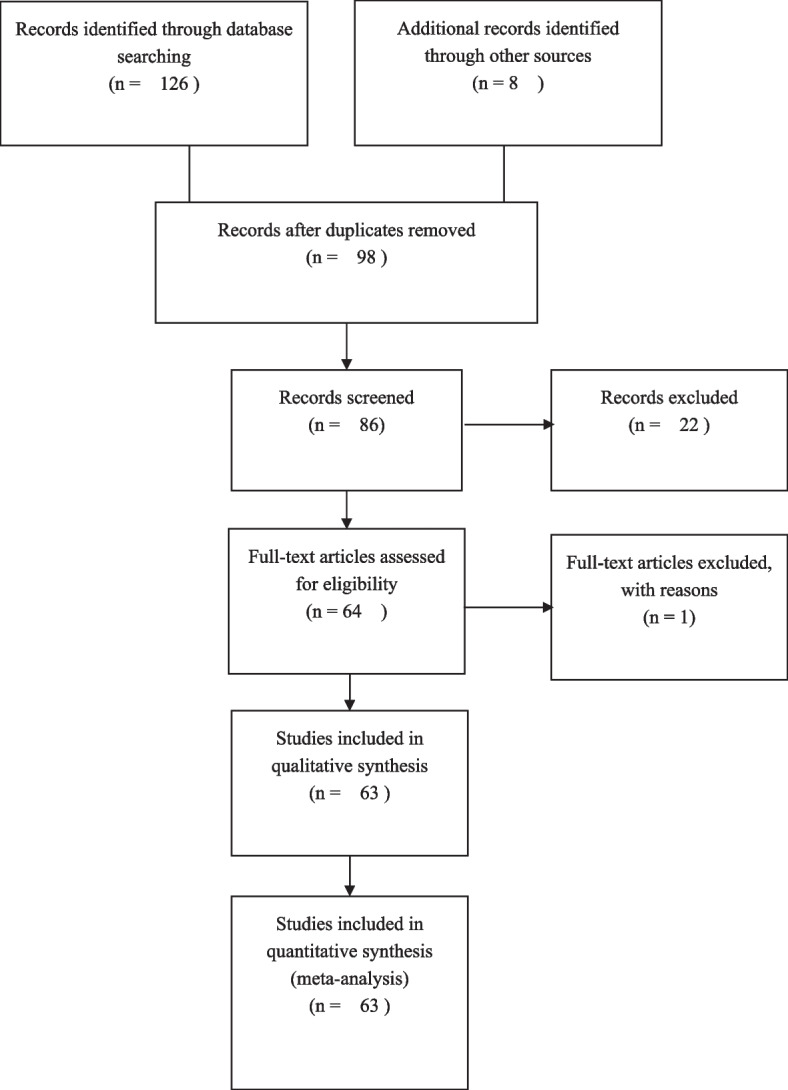
The flow diagram for included and excluded studies

Figure 1 shows the flow diagram detailing the process of trial identification and selection. Finally, 63 articles were eligible for inclusion in this meta-analysis. The major characteristics of the eligible studies were shown in Supplementary Table 1. Overall, data were available for 15 genetic variants of 6 genes, each containing at least 3 independent studies. The detailed information of different gene variants was listed in Table 1. Based on the number of different independent studies of each gene variant (ranging from 3 to 30), the 6 most commonly studied variants were the following: GSTM1 rs366631 (*n* = 30), GSTT1 rs17856199 (*n* = 27), GSTP1 A313G rs1695 (*n* = 19), EPHX T113C rs1051740 (*n* = 24), and EPHX A139G rs2234922 (*n* = 22). According to the ethnicity, 31 studies were performed in Asian, 24 were in Caucasian, and 4 in African. Subgroup analyses were performed according to ethnicity and HWE.

**Table 1:** Information of different variants

Genes	Variant ID	Chr	Risk/Minor alle	SNP Location	Studies No	Case	Control
EPHX T113C	rs1051740	1q42.1	C	Exon 3	24	8618	43100
EPHX A139G	rs2234922	1q42.1	G	Exon 4	22	9298	9109
GSTP1 A313G	rs1695	11q13	G	Exon 5	19	2452	3206
GSTP1 C341T	rs1138272	11q13	T	Exon 6	5	876	1153
GSTM1	rs366631	1p13.3	Null	deletions	30	4443	5103
GSTT1	rs17856199	22q11.2	Null	deletions	27	3370	4011
CAT A-21T	rs7943316	11p13	T	promoter	3	638	1502
CAT C-262T	rs1001179	11p13	T	promoter	5	982	2063
CYP1A1 MspI	rs4646903	15q22-24	C	3'-flanking	8	1122	1380
CYP1A1 A462G	rs1048943	15q22-24	G	Exon 7	5	715	988
CYP 2E1 RsaI	rs2031920	10q24.3	T	5'-flanking	4	308	534
SOD2 Ala 16 Val	rs4880	6q25	C	Exon 2	3	497	395
SOD2 Val 9 Ala	rs179972	6q25	C	Exon 2	3	231	182
SOD3 A213G	rs1799895	4pter-2q1	G	Exon 3	5	1262	1891
HO-1	rs3074372	22q1213	GT repeats	5'-flanking	7	1013	5113

### Meta-analysis results

We used five genetic models in this meta-analysis. The diversity of genetic models helped us to judge the possible inheritance of COPD. Most studies satisfied HWE which means that the control populations have reached a genetic balance, and the data of theses populations survey is credible. In total, 167 meta-analyses, which 64 were overall meta-analyses under the five different genetic models; in subgroup analyses 53 were to ethnicity and 50 in accordance to HWE. The results of the primary meta-analyses and subgroup meta-analyses under the five different genetic models were listed in Supplementary Table 2, Supplementary Table 3 and Supplementary Table 4. Significant associations (*p* < 0.05) with the risk of COPD were found with EPHX T113C, GSTP1, GSTM1, GSTT1, CAT C262T, CYP1A1 MspI, SOD2 Val 9 Ala, SOD3 A213G and HO-1 (Table 2).

**Table 2:** Genetic variants significantly associated with COPD risk in meta-analysis

SNP	R/M	Group	Com	No	OR(95%CI)	*P*	I^2^(%)	Pheterogeneity	Level of Evidence	Venice criteria		
EPHX T113C	C	All	R	22	1.27 [1.06, 1.52]	0.01	59	< 0.01	weak	AAC		0.149
			D	24	1.25 [1.09, 1.43]	< 0.01	65	< 0.01	weak	ABC	2.66	0.014
			CD	23	1.38 [1.13, 1.69]	< 0.01	60	< 0.01	weak	AAC	1.94	0.066
			A	23	1.18 [1.07, 1.30]	< 0.01	66	< 0.01	weak	ABC	2.57	0.018
		Asian	R	11	1.30 [1.03, 1.64]	0.03	41	0.08	moderate	AAB	-0.20	0.847
			D	12	1.41 [1.05, 1.89]	0.02	66	< 0.01	weak	AAC	-0.58	0.575
			CD	11	1.56 [1.27, 1.91]	< 0.01	36	0.11	moderate	AAB	-0.35	0.735
			A	12	1.24 [1.02, 1.49]	0.03	71	< 0.01	weak	AAC	0.36	0.725
		HWE	A	15	1.13 [1.02, 1.25]	0.02	58	< 0.01	weak	AAC	1.91	0.078
EPHX A139G	G	Asian	D	10	0.81 [0.69, 0.96]	0.01	0	0.45	strong	AAA	1.93	0.089
			A	10	0.84 [0.73, 0.96]	0.01	0	0.44	strong	AAA	2.08	0.071
			H	9	0.80 [0.67, 0.95]	0.01	0	0.36	strong	AAA	1.63	0.148
GSTP1 A313G	G	All	R	19	1.54 [1.10, 2.17]	0.01	59	< 0.01	weak	AAC	0.53	0.603
			CD	19	1.54 [1.03, 2.29]	0.03	65	< 0.01	weak	AAC	0.91	0.377
		Asian	R	12	1.94 [1.50, 2.52]	< 0.01	21	0.24	strong	AAA	0.17	0.868
			CD	12	1.94 [1.45, 2.60]	< 0.01	17	0.27	strong	AAA	0.56	0.591
			A	12	1.28 [1.05, 1.55]	0.01	58	< 0.01	weak	AAC	-0.47	0.649
		HWE	R	14	1.71 [1.34, 2.18]	0.01	30	0.14	moderate	AAB	0.46	0.652
			CD	14	1.77 [1.19, 2.62]	< 0.01	45	0.04	moderate	AAB	0.41	0.690
			A	14	1.25 [1.04, 1.51]	0.02	65	< 0.01	weak	AAC	-0.51	0.619
GSTP1 C341T	T	All	D	5	1.96 [1.16, 3.29]	0.01	80	< 0.01	weak	BAC	1.45	0.243
			A	5	1.52 [1.09, 2.13]	0.01	76	< 0.01	weak	BAC	1.61	0.205
			H	5	1.91 [1.16, 3.14]	0.01	76	< 0.01	weak	BAC	1.45	0.243
SNP	R/M	Group	Com	No	OR(95%CI)	P	I^2^(%)	Pheterogeneity	Level of Evidence	Venice criteria		
CAT C262T	T	All	CD	5	0.52 [0.32, 0.84]	< 0.01	17	0.31	moderate	BAA	0.87	0.477
CYP1A1 MspI	C	All	CD	7	1.51 [1.06, 2.16]	0.02	35	0.16	moderate	AAB	-1.20	0.282
		Asian	R	4	1.47 [1.01, 2.14]	0.04	42	0.16	moderate	AAB	-2.14	0.166
		HWE	R	6	1.58 [1.10, 2.27]	0.01	0	0.58	strong	AAA	-1.11	0.328
			CD	6	1.74 [1.19, 2.55]	< 0.01	0	0.48	strong	AAA	-0.82	0.458
CYP1A1 A462G	G	HWE	R	3	2.66 [1.22, 5.77]	0.01	54	0.11	weak	BAC	-1.61	0.354
SOD2 Val 9 Ala	C	ALL	R	3	0.56 [0.35, 0.90]	0.02	55	0.11	weak	BAC	1.13	0.461
		HWE	R	3	0.56 [0.35, 0.90]	0.02	55	0.11	weak	BAC	1.13	0.461
SOD3 A213G	G	ALL	CD	4	3.56 [1.80, 7.06]	< 0.01	51	0.10	weak	BAC	0.13	0.907
GSTM1	Null	All	N	30	1.59 [1.37, 1.86]	< 0.01	64	< 0.01	weak	AAC	0.42	0.676
		Asian	N	17	1.67 [1.31, 2.13]	< 0.01	67	< 0.01	weak	AAC	-1.39	0.184
		Caucasian	N	10	1.36 [1.12, 1.63]	< 0.01	40	0.09	moderate	AAB	0.54	0.607
		African	N	3	1.87 [1.14, 3.07]	0.01	80	< 0.01	weak	AAC	1.08	0.476
GSTT1	Null	All	N	27	1.18 [1.02, 1.37]	0.02	44,	< 0.01	moderate	AAB	2.05	0.051
		Asian	N	16	1.26 [1.01, 1.57]	0.04	58	0.02	weak	AAC	1.80	0.093
GSTM1/GSTT1	Null	All	N	16	1.39[1.15,1.67]	< 0.001	0	0.45	strong	AAA	1.49	0.158
		Caucasian	N	3	1.58[1.09,2.27]	0.01	27	0.25	moderate	BAB	-0.30	0.790
HO-1(Type 1)	GTn	All	R	7	1.66 [1.36, 2.03]	< 0.01	0	0.48	strong	AAA	-0.90	0.411
		Asian	R	5	1.77 [1.38, 2.28]	< 0.01	0	0.68	moderate	BAA	0.55	0.618
HO-1(L Type)		All	R	5	1.69 [1.40, 2.04]	< 0.01	28	0.24	moderate	BAB	-0.63	0.573
		Asian	R	3	1.84 [1.47, 2.30]	< 0.01	0	0.44	moderate	BAA	1.43	0.389

*R/M* risk/minor alle, *COM* comparison, *R* recessive model (GG vs. GA + AA), *D* dominant genetic model (GG + GA vs. AA), *CD* co-dominant model (GG vs. AA), *A* allele model (G *vs.* A), *H* heterozygote model (GA vs. AA), *N* Null *vs.* Present

### Polymorphisms in Epoxide hydrolase 1(EPHX1)

Twenty-four studies (including 8618 COPD cases and 43100 controls) were performed to determine the association between EPHX1 T113C and the risk of COPD. There was a significantly increased risk of COPD under the recessive model ((OR = 1.27, 95% CI = 1.06–1.52, *p* = 0.01), dominant model (OR = 1.25, 95% CI = 1.09–1.43, *p* < 0.01), co-dominant model (OR = 1.38, 95% CI = 1.13–1.69, *p* < 0.01) and allele model (OR = 1.18, 95% CI = 1.07–1.30, *p* < 0.01). In the 24 studies,12 studies were performed in Asians, 10 in Caucasians and 2 in Africans. The EPHX1 T113C C allele could increase the COPD risk in Asians but not in Caucasians. In the analysis stratified in accord with HWE, the pooled OR (OR = 1.13; 95% CI 1.02–1.25, *p* = 0.02) showed a significant association between the EPHX1 T113C allele models and COPD risk.

A total of 22 studies (including 9298 COPD cases and 9109 controls) detected the association between the EPHX1 A139G and COPD risk. In the overall and HWE analyses, the EPHX1 A139G G allele showed no association with COPD risk. In the analysis stratified by ethnicity, the SNP showed decreased risk in Asian populations in dominant model (OR = 0.81, 95% CI = 0.69–0.96, *p* = 0.01), allele model (OR = 0.84, 95% CI = 0.73–0.96, *p* = 0.01), heterozygote model (OR = 0.80, 95% CI = 0.67–0.95, *p* = 0.01).

In summary, EPHX T113C C allele showed increased COPD risk, especially in Asians; while A139G G allele could decrease COPD risk in Asians.

### Polymorphisms in Glutathione S-transferases (GSTs)

Thirty case–control studies met the inclusion criteria for GSTM1 (including 4443 COPD cases and 5103 controls), 27 studies for GSTT1 (including 3376 cases and 4014 controls), and 16 studies for GSTM1-GSTT1 combined effects (including 1708 cases and 1936 controls). Pooled analyses on the association between GSTM1 null/present polymorphism and COPD risk were observed to be significant in overall and subgroup analyses. The individual who carries the GSTM1 null-genotype had an increased COPD risk in overall populations (OR = 1.59, 95% CI = 1.37–1.86, *p* < 0.01). When ethnicity subgroups were considered in the meta-analysis, the results were the same as the overall analysis in Asians, Caucasians and Africans. The GSTT1 null-genotype could statistically increase the COPD risk in overall and Asian populations, but not in Caucasians and Africans. For the GSTM1-GSTT1 interaction, we found that the GSTM1-GSTT1 combined null-genotype was significantly associated with COPD risks in overall populations (OR = 1.39, 95% CI: 1.15–1.67, *p* < 0.01), analysis of racial stratification showed no correlation.

Nineteen studies (including 2452 COPD cases and 3206 controls) evaluated GSTP1 polymorphism with COPD risk. The SNP A313G, the results showed that G allele associated with increased CODP risk in the overall and subgroup analysis. To the ethnical analyses, A313G G allele showed increased COPD risk in Asians, while the Caucasians showed no significant association. To the SNP C341T, 5 articles was included. T allele showed statistical significance in the overall analysis but not in subgroups analyses.

In our meta-analysis, we found that GSTM1 null, GSTT1 null, GATP1 A313G G and C341T T allele were associated with elevated COPD risk.

### Polymorphisms in CAT and CYP

We analyzed the polymorphism of A21T and C262T in CAT to evaluate the COPD risk. There were 3 studies about A21T and 5 studies about C262T, for the subgroup analyses wo could only analyze in accord with HWE. T allele in A21T showed no association with COPD risk. The C262T T allele was associated with decreased COPD risk in co-dominant model (OR = 0.52, 95% CI: 0.32–0.84, *p* < 0.01).

There were 8 studies about CYP1A1 MspI, 5 about CYP1A1 A462G and 4 about CYP 2E1 RsaI. In the overall analyses for three SNPs, MspI polymorphism showed increased COPD risk, and another two SNPs showed no association. Only two studies in accord with HWE for RsaI, so we analyzed MspI and A262G in this subgroup. Results showed that C allele in MspI and G alle in A462G were associated with decreased COPD risk. Only MspI polymorphism was analyzed in the ethnical subgroup, and the C allele showed increased COPD risk in Asian populations.

The results showed that the polymorphisms in CAT C262T, CYP1A1 MspI and CYP1A1 A462G were statistically significant with COPD risk.

### Polymorphisms in SOD and HO-1

We analyzed Val 9 Ala and Ala 16 VAL polymorphisms in SOD2, and A213G in SOD3. The results showed that Val 9 Ala and A213G were associated with COPD risk. The L allele in the HO-1 increased COPD risk in overall and subgroup analyses.

### Sensitivity analysis, publication bias and venice criteria

A sensitivity analysis was performed to evaluate the stability of the individual data to the pooled OR. After sequentially excluding each one of the included studies that assessed the overall relationship between each SNP polymorphism and COPD risk, statistically similar results were obtained, suggesting the results of this meta-analysis were stable. Moreover, publication bias was assessed by Begg’s funnel plots and Egger’s test in the recessive models. Although the shape of the funnel plots appeared asymmetrical in some SNP, the *p* values were all > 0.05 (Table 3), indicating an absence of publication bias in the recessive models. In this meta-analysis, we used Venice criteria to assess credibility of each significant variant. Based on the degree of amount, replication and protection from bias, we identified 4 strong and 6 moderate variants to access the epidemiological evidence associations with the COPD risk.

**Table 3:** Results of publication bias by Begg’s funnel plots and Egger’s test

Genes	Begg's funnel plo	t	P
EPHX T113C	symmetry	1.50	0.149
EPHX A139G	asymmetry	-1016	0.263
GSTP1 A313G	symmetry	0.53	0.603
GSTP1 C341T	symmetry	0.00	0.998
GSTM1	symmetry	0.42	0.676
GSTT1	symmetry	2.05	0.051
CAT A-21T	asymmetry	-2.16	0.276
CAT C-262T	asymmetry	0.41	0.719
CYP1A1 MspI	asymmetry	-1.44	0.209
CYP1A1 A462G	asymmetry	-3.15	0.088
CYP 2E1 RsaI	asymmetry	2.25	0.154
SOD2 Ala 16 Val	asymmetry	-0.73	0.600
SOD2 Val 9 Ala	asymmetry	1.13	0.461
SOD3 A213G	asymmetry	0.25	0.828
HO-1	asymmetry	-0.90	0.411

## Discussion

During the past 20 years, significant advances have been made in the epidemiology, diagnosis, and treatment of COPD, but the etiology and mechanisms of the disease have not been fully elucidated [15]. Genetics and smoking are key factors in the development of COPD. Genome-wide association studies (GWAS) found many COPD risk candidate genes, such as CHRNA3/CHRNA5/IREB2, HHIP, FAM13A, RAB4B, EGLN2, MIA, CYP2A6, SOD3, MMP9, et al. [16]. CYP2A6 and SOD3 are oxidative stress candidate genes. Except of CYP2A6, polymorphisms of many other oxidative stress candidate genes were studied, but the results were controversial. COPD risk with the polymorphisms of some genes [10], GSTM1/GSTT1 [9], GSTP1 [17], HO-1 [11], CYP [12] were evaluated by meta-analysis. Despite all this, our study is the first comprehensive meta-analysis to summarize evidence of associations between oxidative stress-related genes polymorphisms and COPD risk. Although there were studies performed previously to analyze single SNP polymorphism with COPD risk, these studies were conducted more than five years. New analyses are needed to evaluate the candidate genes polymorphism with COPD risk. In this study, we reviewed 63 existing studies on the association between polymorphisms in the oxidative stress genes and COPD risk. Our study analysis 15 variants of 6 genes, and 7 SNPs in GSTP1, CAT, CYP, SOD were first analyses until now.

Epoxide hydrolase1(EPHX1) is an enzyme involved in the first-pass metabolism of highly active epoxide intermediates. Two SNPs affecting the enzyme activity in EPHX1 gene have been widely studied. In vitro studies, tyrosine at codon 113 in exon 3 was replaced by histidine (Tyr113His), which reduced the enzyme activity by > 50%, and the mutant is referred as the “slow” allele. Histidine at codon 139 in exon 4 was replaced by arginine (His139Arg), which increased the enzyme activity by more than 25%, and is referred as the “fast” allele [18]. In this meta-analysis, the “slow” allele was significantly associated with increased COPD risk, and the “fast” allele with decreased COPD risk. GSTs are a superfamily of the enzymes which play an important role in detoxifying various aromatic hydrocarbons. GSTM1 is involved in the metabolism of diol epoxides stemming from policy clicaromatic hydrocarbons (PAHs) and ROS. GSTT1 can detoxify methylating agents, pesticides and many chemicals present in cigarette smoke [19]. The homozygous deletion variant GSTM1 null and GSTT1 null induce the enzymes activity loss [20]. In our study, we found that the null type increased COPD risk in GSTM1 and GSTT1 alone or combined; with the exception of GSTT1 null type in Caucasians and Africans. Enzyme activity is also affected by GSTP1 for replacing isoleucine with valine in exon 5 and alanine with valine in exon 6 [21]. Overall, these mutant in GSTP1 were significantly with increased COPD risk in our study. HO-1 has anti-oxidant role, and the gene is referred as a cytoprotective stress response gene [22]. A dinucleotide repeat (GT)n sequence regulates the heme-degrading enzyme expression, and the long allele (L) could reduce enzyme activity [23]. L refers to GTn ≥ 32 GT repeats, M for 26–31 repeats and S for ≤ 25 GT repeats. Type 1 stands for L type allele. In our study, we found type 1 and L allele with increased COPD risk. Antioxidant enzyme activity is negatively with COPD risk. C allele in EPHX T113C, G allele in GSTP1 A3313G, T allele in GSTP1 C341T, null in GSTM1/GSTT1, L allele in HO-1 affect the enzyme activity, meanwhile, these mutants could increase the COPD risk. G allele in EPHX1 A139G associated with increase enzyme activity, and in our study, we found G allele with decreased COPD risk. Mutants or minor alleles in CYP, CAT and SOD were also associated with enzyme activity. In our study, Increased COPD risk was found in CYP1A1 MspI C allele and SOD3 A213G G allele; and decreased risk in CAT C262T T allele and SOD2 Val 9 Ala C allele. In the study, the polymorphisms of GSTP1 C341T, CAT A21T, CAT C262T, CYP 2E1 RsaI, SOD2 Ala 16 Val, SOD2 Val 9 Ala, SOD3 A213G with COPD risk were firstly analyzed.

Heterogeneity and publication bias determined the reliability of results in a meta-analysis [24]. In some comparisons significant heterogeneity was detected. This may because of the existence of design difference among the included studies and the genetic distribution data of some genes cannot extracted separately. Exclusion of some studies can avoid high heterogeneity; in our study we conducted subgroups analysis in accordance with HWE and ethnicity to exclude some study. The results for publication bias and sensitivity analysis indicated the reliability of our meta-analysis. The Venice criteria is an important method to evaluate the credibility of a meta-analysis. According to the criteria, the degree of amount, replication and protection from bias, and then the level of strengthen was accessed by A, B and C grades. The strong and moderate levels suggested the strengthen of epidemiological association with COPD risk. Even so, additional evidence is necessary for further evaluation, such as gene knockout experiments.

Several potential limitations of this meta-analysis should be discussed. First, since our literature search was conducted only in the selected databases, we might have missed relevant studies deposited in other databases. Second, since we only included published studies written in English, studies in other languages were excluded. Third, most of the included studies were conducted in Caucasian and Asian populations, therefore, the results may only be applicable to these populations. Finally, as there were less than 3 studies on some gene variants, they could not be included in the meta-analysis, so not all oxidative stress related genes variants were included in this study. However, this study also has some clear advantages. First, this study is the first and most comprehensive to assess the relationship between the oxidative stress candidate genes and COPD risk. Second, we performed a subgroup analysis stratified by ethnicity and accordance with HWE to ameliorate high heterogeneity. Third, the scientific search and selection method significantly increased the reality of the meta-analysis. All in all, this meta-analysis has made an important contribution to this field.

## Conclusion

In summary, we performed the first comprehensive meta-analysis to assess the oxidative stress genes with the susceptibility to COPD. Our results confirm that several candidate genes were significantly associated with COPD risk. Future functional studies are needed to confirm the relationship between these candidate genes and COPD. And multi-center GWAS study is required for further assessment especially for the 7 SNPs first analyzed.

### Supplementary Information

**Additional file 1:** **Supplementary Table 1.** The characteristics of the eligible studies. **Supplementary Table 2.** Summary of results from different comparative genetic models. **Supplementary Table 3.** Summary of results of subgroup comparative genetic models. **Supplementary Table 4.** Summary of results from different comparative genetic models in accord to HWE.

**Additional file 2. **

**Additional file 3.**

## Data Availability

The raw data supporting the conclusions of this manuscript will be made available by the authors, without undue reservation, to any qualified researcher. Further enquiries can be directed to the Shujin Guo.
