# Supplementary material for: Association between the oxidative stress gene polymorphism and chronic obstructive pulmonary disease risk: a meta-analysis
Source: BMC Pulm Med. 2023 Oct 10;23:384. doi: 10.1186/s12890-023-02625-y (PMC10566167; doi:10.1186/s12890-023-02625-y)
Supplement: Supplementary file 1 — Additional file 1: Supplementary Table 1. The characteristics of the eligible studies. Supplementary Table 2. Summary of results from different comparative genetic models. Supplementary Table 3. Summary of results of subgroup comparative genetic models. Supplementary Table 4. Summary of results from different comparative genetic models in accord to HWE. [file 12890_2023_2625_MOESM1_ESM.docx]

**Supplementary Table 1：** The characteristics of the eligible studies

| Author[Ref] | Year | Country | Ethnicity | Case | Control | SNP method | Gene | Quality score |
| --- | --- | --- | --- | --- | --- | --- | --- | --- |
| 1. Takeyabu | 2000 | Japan | Asian | 79 | 172 | direct sequencing | EPHX | 7 points |
| 1. Yamada | 2000 | Japan | Asian | 101 | 100 | direct sequencing | HO-1 | 7 points |
| 1. Yim | 2000 | Koreans | Asian | 83 | 76 | PCR-RFLP | GST, EPHX | 8 points |
| 1. Yosgikawa | 2000 | Japan | Asian | 40 | 318 | PCR-RFLP | EPHX | 6 points |
| 1. Rodriguez | 2002 | Spanish | Caucasian | 79 | 146 | PCR‑RFLP | EPHX | 8 points |
| 1. Yim | 2002 | Koreans | Asian | 89 | 94 | PCR‑RFLP | GST | 8 points |
| 1. Budhi | 2003 | Japan | Asian | 63 | 172 | PCR-RFLP | GST, EPHX, CYP, HO-1 | 8 points |
| 1. Cheng | 2004 | China | Asian | 184 | 212 | PCR-RFLP | GST, EPHX | 8 points |
| 1. Xiao | 2004 | China | Asian | 100 | 100 | PCR-RFLP | GST, EPHX | 8 points |
| 1. Gaspar | 2004 | Brazil | Caucasian | 75 | 187 | Multiplex PCR | GST, CYP | 8 points |
| 1. Yanchina | 2004 | Russia | Caucasian | 72 | 77 | PCR | GST | 6 points |
| 1. Hersh | 2005 | USA | Caucasian | 304 | 441 | TaqMan | GST | 7 points |
| 1. Park | 2005 | USA | Caucasian | 131 | 262 | PCR-RFLP | EPHX | 8 points |
| 1. Rodriguez | 2005 | Spanish | Caucasian | 98 | 267 | Real-time PCR | GST | 8 points |
| 1. Brøgger | 2006 | Norway | Caucasian | 244 | 248 | TaqMan | EPHX | 9 points |
| 1. Fu | 2006 | China | Asian | 256 | 266 | PCR-RFLP | EPHX， HO-1 | 7 points |
| 1. Matheson | 2006 | Australia | Caucasian | 72 | 220 | ARMS | EPHX | 6 points |
| 1. Calikoglu | 2006 | Turkish | Caucasian | 149 | 150 | Real-time PCR | GST | 9 points |
| 1. Young | 2006 | European | Caucasian | 230 | 210 | PCR-RFLP | CAT, SOD | 8 points |
| 1. Vibhuti | 2007 | India | Asian | 202 | 136 | PCR-RFLP | GST, EPHX | 8 points |
| 1. Chan-Yeung | 2007 | China | Asian | 163 | 163 | Multiplex PCR | GST | 9 points |
| 1. Korytina | 2007 | Russia | Caucasian | 319 | 418 | PCR–PDRF | CYP | 8 points |
| 1. Mak | 2007 | China | Asian | 165 | 165 | PCR-RFLP | CAT, SOD | 9 points |
| 1. Chappell | 2008 | European | Caucasian | 1017 | 912 | TaqMan | EPHX | 8 points |
| 1. Židzik J | 2008 | Slovakia | Caucasian | 217 | 160 | PCR-RFLP | GST, EPHX | 9 points |
| 1. Joyce | 2008 | Dutch | Caucasian | 102 | 20 | PCR | SOD | 8 points |
| 1. Faramawy | 2009 | Egypt | African | 34 | 34 | Multiplex PCR | GST | 5 points |
| 1. Cheng SL | 2009 | China | Asian | 184 | 212 | PCR-RFLP | CYP | 8 points |
| 1. Korytina | 2009 | Bashkortostan | Mixed | 320 | 585 | PCR-RFLP | GST, CAT, SOD | 8 points |
| 1. Penyige | 2010 | Hungarian | Caucasian | 272 | 301 | TaqMan | EPHX | 7 points |
| 1. Chen CZ | 2010 | China | Asian | 105 | 103 | PCR-RFLP | EPHX | 8 points |
| 1. Lakhdar | 2010 | Tunisia | African | 234 | 182 | Multiplex PCR | GST, EPHX | 9 points |
| 1. Mehrotra | 2010 | India | Asian | 50 | 50 | Multiplex PCR | GST | 7 points |
| 1. Vibhuti | 2010 | India | Asian | 210 | 136 | PCR-RFLP | CYP | 8 points |
| 1. Sørheim | 2010 | USA | Caucasian | 389 | 472 | Taqman assays | SOD | 9 points |
| 1. Pietras | 2010 | Poland | Caucasian | 162 | 63 | PCR-RFLP | SOD | 7 points |
| 1. Lee J | 2011 | Denmark | Caucasian | 4127 | 37964 | TaqMan | EPHX | 7 points |
| 1. Putra | 2011 | Japan | Asian | 48 | 172 | PCR-RFLP | GST, EPHX, CYP, HO-1 | 8 points |
| 1. Yechshzhanov | 2011 | Kazakhstan | Asian | 60 | 119 | PCR-RFLP | GST | 6 points |
| 1. Shukla | 2011 | India | Asian | 204 | 208 | Multiplex PCR | GST | 8 points |
| 1. Young | 2011 | New Zealand | Caucasian | 669 | 488 | Taqman | GST | 9 points |
| 1. Thakur | 2011 | India | Asian | 200 | 200 | Multiplex PCR | GST | 7 points |
| 1. Bose | 2012 | India | Asian | 23 | 70 | PCR-RFLP | GST | 7 points |
| 1. Matokanović | 2012 | Croatia | Caucasian | 130 | 90 | PCR-RFLP | HO-1 | 7 points |
| 1. Zuntar | 2014 | Croatia | Caucasian | 30 | 60 | Multiplex PCR | GST | 7 points |
| 1. Begum | 2014 | Indian | Asian | 250 | 250 | Multiplex PCR | GST | 8 points |
| 1. Dey | 2014 | India | Asian | 70 | 85 | Multiplex PCR | GST | 7 points |
| 1. Yang | 2014 | China | Asian | 101 | 80 | PCR-RFLP | CYP | 9 points |
| 1. Natsuko | 2014 | Japan | Asian | 265 | 1076 | TaqMan | CAT | 7 points |
| 1. Stankovic | 2015 | Serbia | Caucasian | 122 | 100 | PCR-RFLP | GST, EPHX, CYP | 7 points |
| 1. Gandhi | 2015 | India | Asian | 32 | 19 | Multiplex PCR | GST, SOD | 8 points |
| 1. El Wahsh | 2015 | Egypt | African | 146 | 130 | Multiplex PCR | GST, EPHX | 6 points |
| 1. Zivka | 2016 | Serbia | Caucasian | 71 | 153 | PCR-RFLP | GST, EPHX | 6 points |
| 1. Khan | 2016 | India | Asian | 186 | 160 | PCR-RFLP | GST | 8 points |
| 1. Akparova | 2017 | Kazakhstan | Asian | 55 | 52 | PCR | EPHX | 7 points |
| 1. Arpaci | 2018 | Turkey | Caucasian | 100 | 100 | PCR | CAT, SOD | 7 points |
| 1. Cao | 2017 | China | Asian | 33 | 33 | PCR | GST | 8 points |
| 1. Du | 2019 | China | Asina | 150 | 150 | PCR-SBT | GST, SOD, HO-1 | 7 points |
| 1. Anes | 2019 | Tunisia | African | 143 | 216 | PCR-RFLP | CAT | 7 points |
| 1. Ma | 2020 | China | Asina | 441 | 192 | PCR | EPHX | 8 points |
| 1. Würtz | 2020 | Danish | Caucasian | 150 | 150 | PCR | HO-1 | 7 points |
| 1. Ganbold | 2021 | Mongolia | Asina | 181 | 292 | PCR | GST、EPHX | 7 points |
| 1. Tacheva | 2022 | Bulgaria | Caucasian | 152 | 131 | PCR | GST | 7 points |

**Supplementary Table 2**: Summary of results from different comparative genetic models

| SNP | R/M | Com | No | OR(95%CI) | *P* | I^2^(%) (P^*^) | Mo | SNP | R/M | Com | No | OR(95%CI) | *P* | I^2^(%) (P^*^) | Mo |
| --- | --- | --- | --- | --- | --- | --- | --- | --- | --- | --- | --- | --- | --- | --- | --- |
| EPHX, | C | R | 22 | **1.27 [1.06, 1.52]** | **0.01** | 59,＜0.01 | R | EPHX | G | R | 20 | 1.06 [0.92, 1.23] | 0.42 | 12,0.30 | F |
| T113C |  | D | 24 | **1.25 [1.09, 1.43]** | **＜0.01** | 65,＜0.01 | R | A139G |  | D | 22 | 1.01 [0.95, 1.07] | 0.83 | 20,0.20 | F |
|  |  | CD | 23 | **1.38 [1.13, 1.69]** | **＜0.01** | 60,＜0.01 | R |  |  | CD | 20 | 1.06 [0.91, 1.23] | 0.44 | 12,0.31 | F |
|  |  | A | 23 | **1.18 [1.07, 1.30]** | **＜0.01** | 66,＜0.01 | R |  |  | A | 21 | 1.01 [0.95, 1.06] | 0.80 | 22,0.18 | F |
|  |  | H | 22 | 1.17 [1.00, 1.36] | 0.05 | 67,＜0.01 | R |  |  | H | 20 | 0.93 [0.83, 1.03] | 0.17 | 33,0.08 | R |
|  |  |  |  |  |  |  |  |  |  |  |  |  |  |  |  |
| GSTP1 | G | R | 19 | **1.54 [1.10, 2.17]** | **0.01** | 59,＜0.01 | R | GSTP1 | T | R | 5 | 1.31 [0.92, 1.84] | 0.13 | 30,0.22 | F |
| A313G |  | D | 19 | 1.07 [0.84, 1.35] | 0.59 | 74,＜0.01 | R | C341T |  | D | 5 | **1.96 [1.16, 3.29]** | **0.01** | 80,＜0.01 | R |
|  |  | CD | 19 | **1.54 [1.03, 2.29]** | **0.03** | 65,＜0.01 | R |  |  | CD | 5 | 2.00 [0.88, 4.57] | 0.10 | 66,＜0.01 | R |
|  |  | A | 19 | 1.14 [0.94, 1.40] | 0.18 | 79,＜0.01 | R |  |  | A | 5 | **1.52 [1.09, 2.13]** | **0.01** | 76,＜0.01 | R |
|  |  | H | 19 | 0.98 [0.79, 1.22] | 0.87 | 67,＜0.01 | R |  |  | H | 5 | **1.91 [1.16, 3.14]** | **0.01** | 76,＜0.01 | R |
|  |  |  |  |  |  |  |  |  |  |  |  |  |  |  |  |
| CAT | T | R | 3 | 0.91 [0.68, 1.20] | 0.49 | 0,0.42 | F | CAT | T | R | 5 | 0.81 [0.29, 2.26] | 0.69 | 67,0.03 | R |
| A21T |  | D | 3 | 0.90 [0.73, 1.10] | 0.30 | 0,0.74 | F | C262T |  | D | 5 | 1.00 [0.80, 1.26] | 0.98 | 13,0.33 | F |
|  |  | CD | 3 | 0.86 [0.63, 1.18] | 0.35 | 0.0.39 | F |  |  | CD | 5 | **0.52 [0.32, 0.84]** | **＜0.01** | 17,0.31 | F |
|  |  | A | 3 | 0.92 [0.80, 1.07] | 0.27 | 0.0.52 | F |  |  | A | 5 | 0.92 [0.77, 1.10] | 0.37 | 0.0.72 | F |
|  |  | H | 3 | 0.91 [0.73, 1.12] | 0.37 | 0,0.92 | F |  |  | H | 5 | 1.10 [0.87, 1.39] | 0.41 | 49,0.10 | F |
|  |  |  |  |  |  |  |  |  |  |  |  |  |  |  |  |
| CYP1A1 | C | R | 7 | 1.38 [0.98, 1.93] | 0.06 | 26,0.23 | F | CYP1A1 | G | R | 4 | 1.22 [0.27, 5.45] | 0.80 | 59,0.06 | R |
| MspI |  | D | 8 | 1.12 [0.81, 1.53] | 0.50 | 65,＜0.01 | R | A462G |  | D | 5 | 1.02 [0.66, 1.57] | 0.93 | 61,0.04 | R |
|  |  | CD | 7 | **1.51 [1.06, 2.16]** | **0.02** | 35,0.16 | F |  |  | CD | 4 | 1.13 [0.23, 5.49] | 0.88 | 62,0.05 | R |
|  |  | A | 7 | 1.15 [0.91, 1.45] | 0.26 | 59,0.02 | F |  |  | A | 4 | 1.09 [0.62, 1.92] | 0.76 | 78,＜0.01 | R |
|  |  | H | 7 | 1.17 [0.83, 1.65] | 0.38 | 66,＜0.01 | R |  |  | H | 4 | 1.01 [0.75, 1.36] | 0.94 | 52,0.10 | R |
| SNP | R/M | COM | No | OR(95%CI) | *P* | I^2^(%) (P^*^) | Mo | SNP | R/M | COM | No | OR(95%CI) | *P* | I^2^(%) (P^*^) | Mo |
| CYP 2E1 | T | R | 3 | 0.50 [0.08, 3.10] | 0.46 | 0,0.64 | F | SOD2 | C | R | 3 | 1.02 [0.68, 1.52] | 0.93 | 0,0.94 | F |
| RsaI |  | D | 4 | 1.19 [0.81, 1.75] | 0.37 | 44,0.15 | F | Ala 16 Val |  | D | 3 | 1.11 [0.80, 1.53] | 0.54 | 20,0.29 | F |
|  |  | CD | 3 | 1.38 [0.57, 3.37] | 0.48 | 60,0.08 | R |  |  | CD | 3 | 0.94 [0.57, 1.55] | 0.81 | 0,0.87 | F |
|  |  | A | 3 | 1.69 [0.59, 4.84] | 0.33 | 64,0.06 | R |  |  | A | 3 | 1.05 [0.85, 1.31] | 0.64 | 9,0.33 | F |
|  |  | H | 3 | 0.48 [0.08, 3.01] | 0.44 | 0,0.68 | F |  |  | H | 3 | 1.11 [0.80, 1.56] | 0.53 | 20,0.28 | F |
|  |  |  |  |  |  |  |  |  |  |  |  |  |  |  |  |
| SOD2 | C | R | 3 | **0.56 [0.35, 0.90]** | **0.02** | 55,0.11 | F | SOD3 | G | R | 4 | 2.77 [0.70, 10.95] | 0.15 | 53,0.09 | R |
| Val 9 Ala |  | D | 3 | 0.68 [0.21, 2.23] | 0.53 | 82,＜0.01 | R | A213G |  | D | 4 | 1.43 [0.76, 2.69] | 0.27 | 81,＜0.01 | R |
|  |  | CD | 3 | 0.53 [0.13, 2.24] | 0.39 | 80,＜0.01 | R |  |  | CD | 4 | **3.56 [1.80, 7.06]** | **＜0.01** | 51,0.10 | R |
|  |  | A | 3 | 0.78 [0.35, 1.76] | 0.55 | 85,＜0.01 | R |  |  | A | 4 | 1.33 [0.77, 2.27] | 0.30 | 79,＜0.01 | R |
|  |  | H | 3 | 0.74 [0.27, 2.06] | 0.57 | 72,0.03 | R |  |  | H | 4 | 1.31 [0.67, 2.55] | 0.43 | 81,＜0.01 | R |
|  |  |  |  |  |  |  |  |  |  |  |  |  |  |  |  |
| GSTM1 | Null | N | 30 | **1.59 [1.37, 1.86]** | **＜0.01** | 64,＜0.01 | R | GSTTI | Null | N | 27 | **1.18 [1.02, 1.37]** | **0.02** | 44,＜0.01 | R |
| GSTM1/GSTT1 | Null | N | 16 | **1.39[1.15,1.67]** | **<0.001** | 0,0.45 | F |  |  |  |  |  |  |  |  |
|  |  |  |  |  |  |  |  |  |  |  |  |  |  |  |  |
| HO-1(type 1) | GTn | R | 7 | **1.66 [1.36, 2.03]** | **＜0.01** | 0,0.48 | F | HO-1 (L type) | GTn | R | 5 | **1.69 [1.40, 2.04]** | **＜0.01** | 28,0.24 | F |

R/M: risk/minor alle; COM: comparison; R: recessive model (GG vs. GA+AA); D: dominant genetic model (GG+GA vs. AA); CD: co-dominant model (GG vs. AA); A: allele model (G *vs.* A); H: heterozygote model (GA vs. AA); N: Null *vs.* Present; Mo: model; R: random; F: fixed; P^*^: Pheterogeneity

**Supplementary Table 3**: Summary of results of subgroup comparative genetic models

| SNP | Ethnicity | Com | No | OR(95%CI) | *P* | I^2^(%) (P^*^) | SNP | Ethnicity | Com | No | OR(95%CI) | *P* | I^2^(%)(P^*^) |
| --- | --- | --- | --- | --- | --- | --- | --- | --- | --- | --- | --- | --- | --- |
| EPHX1 | Asian | R | 11 | **1.30 [1.03, 1.64]** | **0.03** | 41,0.08 | EPHX1 | Asian | R | 9 | 0.95 [0.63, 1.43] | 0.80 | 0,0.63 |
| T113C |  | D | 12 | **1.41 [1.05, 1.89]** | **0.02** | 66,＜0.01 | A139G |  | D | 10 | **0.81 [0.69, 0.96]** | **0.01** | 0.0.45 |
|  |  | CD | 11 | **1.56 [1.27, 1.91]** | **＜0.01** | 36,0.11 |  |  | CD | 9 | 0.88 [0.59, 1.34] | 0.56 | 0.0.60 |
|  |  | A | 12 | **1.24 [1.02, 1.49]** | **0.03** | 71,＜0.01 |  |  | A | 10 | **0.84 [0.73, 0.96]** | **0.01** | 0,0.44 |
|  |  | H | 11 | 1.31 [0.91, 1.90] | 0.15 | 75,＜0.01 |  |  | H | 9 | **0.80 [0.67, 0.95]** | **0.01** | 0,0.36 |
|  | Caucasian | R | 9 | 1.17 [0.86, 1.59] | 0.31 | 70,＜0.01 |  | Caucasian | R | 9 | 0.93 [0.79, 1.09] | 0.38 | 0,0.50 |
|  |  | D | 10 | 1.02 [0.96, 1.08] | 0.48 | 7,0.38 |  |  | D | 10 | 0.95 [0.89, 1.02] | 0.16 | 0,0.87 |
|  |  | CD | 9 | 1.17 [0.87, 1.57] | 0.30 | 64,＜0.01 |  |  | CD | 9 | 0.92 [0.78, 1.08] | 0.29 | 0,0.57 |
|  |  | A | 9 | 1.02 [0.98, 1.07] | 0.27 | 37,0.12 |  |  | A | 9 | 0.96 [0.90, 1.01] | 0.12 | 0.0.90 |
|  |  | H | 9 | 1.00 [0.94, 1.06] | 0.97 | 10,0.35 |  |  | H | 9 | 0.96 [0.89, 1.03] | 0.21 | 0,0.74 |
|  |  |  |  |  |  |  |  |  |  |  |  |  |  |
| GSTP1 | Asian | R | 12 | **1.94 [1.50, 2.52]** | **＜0.01** | 21,024 | CYP1A1 | Asian | R | 4 | **1.47 [1.01, 2.14]** | **0.04** | 42,0.16 |
| A313G |  | D | 12 | 1.18 [0.92, 1.51] | 0.18 | 55,0.01 | MspI |  | D | 5 | 1.27 [0.81, 1.99] | 0.31 | 67,0.02 |
|  |  | CD | 12 | **1.94 [1.45, 2.60]** | **＜0.01** | 17,0.27 |  |  | CD | 4 | 1.61 [0.86, 3.01] | 0.14 | 52,0.10 |
|  |  | A | 12 | **1.28 [1.05, 1.55]** | **0.01** | 58,＜0.01 |  |  | A | 4 | 1.26 [0.93, 1.71] | 0.14 | 65,0.14 |
|  |  | H | 12 | 1.06 [0.81, 1.37] | 0.68 | 57,＜0.01 |  |  | H | 4 | 1.29 [0.80, 2.06] | 0.30 | 69,0.02 |
|  | Caucasian | R | 5 | 0.92 [0.43, 1.97] | 0.83 | 69,0.01 |  | Caucasian | R | 3 | 1.00 [0.44, 2.27] | 0.99 | 21,0.28 |
|  |  | D | 5 | 0.92 [0.53, 1.58] | 0.76 | 79,＜0.01 |  |  | D | 3 | 0.87 [0.68, 1.11] | 0.27 | 9,0.33 |
|  |  | CD | 5 | 0.93 [0.36, 2.36] | 0.87 | 77,＜0.01 |  |  | CD | 3 | 0.98 [0.43, 2.25] | 0.97 | 3,0.36 |
|  |  | A | 5 | 0.94 [0.58, 1.52] | 0.81 | 85,＜0.01 |  |  | A | 3 | 0.92 [0.71, 1.18] | 0.51 | 0,0.39 |
|  |  | H | 5 | 0.91 [0.58, 1.42] | 0.68 | 65,0.02 |  |  | H | 3 | 1.02 [0.60, 1.73] | 0.95 | 61,0.08 |
|  |  |  |  |  |  |  |  |  |  |  |  |  |  |
| HO-1 | Asian | R | 5 | **1.77 [1.38, 2.28]** | **＜0.01** | **0,0.68** | HO-1 | Asian | R | 3 | **1.84 [1.47, 2.30]** | **＜0.01** | 0,0.44 |
| SNP | Ethnicity | Com | No | OR(95%CI) | *P* | I^2^(%) (P^*^) | SNP | Ethnicity | Com | No | OR(95%CI) | *P* | I^2^(%)(P^*^) |
| GSTP1 | Asian | R | 3 | 1.37 [0.71, 2.66] | 0.35 | 59,0.09 | GSTM1 | Asian | N | 17 | **1.67 [1.31, 2.13]** | **＜0.01** | 67,＜0.01 |
| C341T |  | D | 3 | 1.76 [0.84, 3.69] | 0.14 | 86,＜0.01 |  | Caucasian | N | 10 | **1.36 [1.12, 1.63]** | **＜0.01** | 40,0.09 |
|  |  | CD | 3 | 1.90 [0.68, 5.31] | 0.22 | 79,＜0.01 |  | African | N | 3 | **1.87 [1.14, 3.07]** | **0.01** | 80,＜0.01 |
|  |  | A | 3 | 1.43 [0.86, 2.39] | 0.17 | 85,＜0.01 | GSTT1 | Asian | N | 16 | **1.26 [1.01, 1.57]** | **0.04** | 58,0.02 |
|  |  | H | 3 | 1.68 [0.86, 3.29] | 0.13 | 81,＜0.01 |  | Caucasian | N | 7 | 1.12 [0.88, 1.43] | 0.35 | 26,0.23 |
|  |  |  |  |  |  |  |  | African | N | 3 | 1.18 [0.87, 1.62] | 0.29 | 0,0.94 |
|  |  |  |  |  |  |  |  |  |  |  |  |  |  |
| GSTM1/GSTT1 | Asian | N | 9 | 1.28[1.00,1.64] | 0.05 | 6,0.38 |  |  |  |  |  |  |  |
|  | Caucasian | N | 3 | **1.58[1.09,2.27]** | **0.01** | 27,0.25 |  |  |  |  |  |  |  |

COM: comparison; R: recessive model (GG vs. GA+AA); D: dominant genetic model (GG+GA vs. AA); CD: co-dominant model (GG vs. AA); A: allele model (G *vs.* A); H: heterozygote model (GA vs. AA); N: Null *vs.* Present; P^*^: Pheterogeneity

**Supplementary Table 4**: Summary of results from different comparative genetic models in accord to HWE

| SNP | R/M | Com | No | OR(95%CI) | *P* | I^2^(%) (P^*^) | Mo | SNP | R/M | Com | No | OR(95%CI) | *P* | I^2^(%) (P^*^) | Mo |
| --- | --- | --- | --- | --- | --- | --- | --- | --- | --- | --- | --- | --- | --- | --- | --- |
| EPHX, | C | R | 15 | 1.25 [1.00, 1.56] | 0.05 | 64,＜0.01 | R | EPHX | G | R | 18 | 1.07 [0.92, 1.25] | 0.36 | 17,0.25 | F |
| T113C |  | D | 15 | 1.09 [0.97, 1.22] | 0.14 | 36,0.08 | R | A139G |  | D | 18 | 1.01 [0.94, 1.07] | 0.85 | 29,0.12 | F |
|  |  | CD | 15 | 1.25 [1.00, 1.57] | 0.06 | 57,＜0.01 | R |  |  | CD | 18 | 1.07 [0.92, 1.25] | 0.38 | 18,0.24 | F |
|  |  | A | 15 | **1.13 [1.02, 1.25]** | **0.02** | 58,＜0.01 | R |  |  | A | 18 | 1.01 [0.96, 1.07] | 0.63 | 25,0.16 | F |
|  |  | H | 15 | 1.04 [0.92, 1.18] | 0.51 | 35,0.09 | R |  |  | H | 18 | 0.92 [0.82, 1.03] | 0.13 | 32,0.09 | R |
|  |  |  |  |  |  |  |  |  |  |  |  |  |  |  |  |
| GSTP1 | G | R | 14 | **1.71 [1.34, 2.18]** | **0.01** | 30，0.14 | F | GSTP1 | T | R | 3 | 0.88 [0.52, 1.50] | 0.65 | 0,0.53 | F |
| A313G |  | D | 14 | 1.18 [0.95, 1.46] | 0.14 | 57,＜0.01 | R | C341T |  | D | 3 | 1.66 [0.80, 3.46] | 0.13 | 83,＜0.01 | R |
|  |  | CD | 14 | **1.77 [1.19, 2.62]** | **＜0.01** | 45,0.04 | R |  |  | CD | 3 | 1.42 [0.42, 4.80] | 0.57 | 71,0.03 | R |
|  |  | A | 14 | **1.25 [1.04, 1.51]** | **0.02** | 65,＜0.01 | R |  |  | A | 3 | 1.35 [0.84, 2.16] | 0.22 | 79,0.01 | R |
|  |  | H | 14 | 1.08 [0.89, 1.32] | 0.43 | 44,0.04 | R |  |  | H | 3 | 1.74 [0.82, 3.66] | 0.15 | 82,＜0.01 | R |
|  |  |  |  |  |  |  |  |  |  |  |  |  |  |  |  |
| CAT | T | R | 3 | 0.91 [0.68, 1.20] | 0.49 | 0,0.42 | F | CAT | T | R | 4 | 1.23 [0.71, 2.13] | 0.46 | 0,0.64 | F |
| A21T |  | D | 3 | 0.90 [0.73, 1.10] | 0.30 | 0,0.74 | F | C262T |  | D | 4 | 0.83 [0.57, 1.20] | 0.32 | 0,0.39 | F |
|  |  | CD | 3 | 0.86 [0.63, 1.18] | 0.35 | 0.0.39 | F |  |  | CD | 4 | 0.81 [0.38, 1.71] | 0.58 | 0,0.40 | F |
|  |  | A | 3 | 0.92 [0.80, 1.07] | 0.27 | 0.0.52 | F |  |  | A | 4 | 0.94 [0.70, 1.25] | 0.66 | 0.0.56 | F |
|  |  | H | 3 | 0.91 [0.73, 1.12] | 0.37 | 0,0.92 | F |  |  | H | 4 | 0.79 [0.54, 1.16] | 0.22 | 0,0.40 | F |
|  |  |  |  |  |  |  |  |  |  |  |  |  |  |  |  |
| CYP1A1 | C | R | 6 | **1.58 [1.10, 2.27]** | **0.01** | 0,0.58 | F | CYP1A1 | G | R | 3 | **2.66 [1.22, 5.77]** | **0.01** | 54,0.11 | F |
| MspI |  | D | 6 | 1.26 [0.87, 1.82] | 0.23 | 70,＜0.01 | R | A462G |  | D | 3 | 1.03 [0.52, 2.07] | 0.93 | 79,＜0.01 | R |
|  |  | CD | 6 | **1.74 [1.19, 2.55]** | **＜0.01** | 0,0.48 | F |  |  | CD | 3 | 1.72 [0.34, 8.61] | 0.51 | 62,0.07 | R |
|  |  | A | 6 | 1.21 [0.94, 1.56] | 0.13 | 57,0.04 | R |  |  | A | 3 | 1.13 [0.54, 2.34] | 0.74 | 85,＜0.01 | R |
|  |  | H | 6 | 1.21 [0.81, 1.80] | 0.35 | 71,＜0.01 | R |  |  | H | 3 | 0.96 [0.69, 1.32] | 0.79 | 63,0.07 | R |
| SNP | R/M | COM | No | OR(95%CI) | *P* | I^2^(%) (P^*^) | Mo | SNP | R/M | COM | No | OR(95%CI) | *P* | I^2^(%) (P^*^) | Mo |
| SOD2 | C | R | 3 | **0.56 [0.35, 0.90]** | **0.02** | 55,0.11 | F | SOD2 | C | R | 3 | 1.02 [0.68, 1.52] | 0.93 | 0,0.94 | F |
| Val 9 Ala |  | D | 3 | 0.68 [0.21, 2.23] | 0.53 | 82,＜0.01 | R | Ala 16 Val |  | D | 3 | 1.11 [0.80, 1.53] | 0.54 | 20,0.29 | F |
|  |  | CD | 3 | 0.53 [0.13, 2.24] | 0.39 | 80,＜0.01 | R |  |  | CD | 3 | 0.94 [0.57, 1.55] | 0.81 | 0,0.87 | F |
|  |  | A | 3 | 0.78 [0.35, 1.76] | 0.55 | 85,＜0.01 | R |  |  | A | 3 | 1.05 [0.85, 1.31] | 0.64 | 9,0.33 | F |
|  |  | H | 3 | 0.74 [0.27, 2.06] | 0.57 | 72,0.03 | R |  |  | H | 3 | 1.11 [0.80, 1.56] | 0.53 | 20,0.28 | F |

COM: comparison; R: recessive model (GG vs. GA+AA); D: dominant genetic model (GG+GA vs. AA); CD: co-dominant model (GG vs. AA); A: allele model (G *vs.* A); H: heterozygote model (GA vs. AA); N: Null *vs.* Present; P^*^: Pheterogeneity

Supplementary references

1. Takeyabu K, Yamaguchi E, Suzuki I, et al. Gene polymorphism for microsomal epoxide hydrolase and susceptibility to emphysema in a Japanese population. The European respiratory journal. 2000;15(5):891-894.

2. Yamada N, Yamaya M, Okinaga S, et al. Microsatellite polymorphism in the heme oxygenase-1 gene promoter is associated with susceptibility to emphysema. American journal of human genetics. 2000;66(1):187-195.

3. Yim JJ, Park GY, Lee CT, et al. Genetic susceptibility to chronic obstructive pulmonary disease in Koreans: combined analysis of polymorphic genotypes for microsomal epoxide hydrolase and glutathione S-transferase M1 and T1. Thorax. 2000;55(2):121-125.

4. Yoshikawa M, Hiyama K, Ishioka S, et al. Microsomal epoxide hydrolase genotypes and chronic obstructive pulmonary disease in Japanese. International journal of molecular medicine. 2000;5(1):49-53.

5. Rodriguez F, Jardi R, Costa X, et al. Detection of polymorphisms at exons 3 (Tyr113-->His) and 4 (His139-->Arg) of the microsomal epoxide hydrolase gene using fluorescence PCR method combined with melting curves analysis. Analytical biochemistry. 2002;308(1):120-126.

6. Yim JJ, Yoo CG, Lee CT, et al. Lack of association between glutathione S-transferase P1 polymorphism and COPD in Koreans. Lung. 2002;180(2):119-125.

7. Budhi A, Hiyama K, Isobe T, et al. Genetic susceptibility for emphysematous changes of the lung in Japanese. International journal of molecular medicine. 2003;11(3):321-329.

8. Cheng SL, Yu CJ, Chen CJ, et al. Genetic polymorphism of epoxide hydrolase and glutathione S-transferase in COPD. The European respiratory journal. 2004;23(6):818-824.

9. Gaspar P, Moreira J, Kvitko K, et al. CYP1A1, CYP2E1, GSTM1, GSTT1, GSTP1, and TP53 polymorphisms: do they indicate susceptibility to chronic obstructive pulmonary disease and non-small-cell lung cancer? .Human and Medical Genetics. 2004;27(2):133–138.

10. Xiao D, Wang C, Du MJ, et al. Relationship between polymorphisms of genes encoding microsomal epoxide hydrolase and glutathione S-transferase P1 and chronic obstructive pulmonary disease. Chinese medical journal. 2004;117(5):661-667.

11. Yanchina ED, Ivchik TV, Shvarts EI, et al. Gene-gene interactions between glutathione-s transferase M1 and matrix metalloproteinase 9 in the formation of hereditary predisposition to chronic obstructive pulmonary disease. Bull Exp Biol Med. 2004;137(1):64-66.

12. Rodriguez F, de la Roza C. Jardi R, et al. Glutathione S-transferase P1 and lung function in patients with alpha1-antitrypsin deficiency and COPD .Chest. 2005:127(5):1537-1543..

13. Hersh CP, Demeo DL, Lange C, et al. Attempted replication of reported chronic obstructive pulmonary disease candidate gene associations. American journal of respiratory cell and molecular biology. 2005;33(1):71-78.

14. Brøgger J, Steen VM, Eiken HG, et al. Genetic association between COPD and polymorphisms in TNF, ADRB2 and EPHX1. The European respiratory journal. 2006;27(4):682-688.

15. Calikoglu M, Tamer L, Ates Aras N, et al. The association between polymorphic genotypes of glutathione S-transferases and COPD in the Turkish population. Biochemical genetics. 2006;44(7-8):307-319.

16. Matheson MC, Raven J, Walters EH, et al. Microsomal epoxide hydrolase is not associated with COPD in a community-based sample. Human biology. 2006;78(6):705-717.

17. Young RP, Hopkins R, Black PN, et al. Functional variants of antioxidant genes in smokers with COPD and in those with normal lung function. Thorax. 2006;61(5):394-399.

18. Chan-Yeung M, Ho SP, Cheung AH, et al. Polymorphisms of glutathione S-transferase genes and functional activity in smokers with or without COPD. The international journal of tuberculosis and lung disease : the official journal of the International Union against Tuberculosis and Lung Disease. 2007;11(5):508-514.

19. Fu WP, Sun C, Dai LM, et al. Relationship between COPD and polymorphisms of HOX-1 and mEPH in a Chinese population. Oncology reports. 2007;17(2):483-488.

20. Mak JC, Ho SP, Yu WC, et al. Polymorphisms and functional activity in superoxide dismutase and catalase genes in smokers with COPD. The European respiratory journal. 2007;30(4):684-690.

21. Vibhuti A, Arif E, Deepak D, et al. Genetic polymorphisms of GSTP1 and mEPHX correlate with oxidative stress markers and lung function in COPD. Biochemical and biophysical research communications. 2007;359(1):136-142.

22. Park JY, Chen L, Wadhwa N, Tockman MS. Polymorphisms for microsomal epoxide hydrolase and genetic susceptibility to COPD. Int J Mol Med. 2005;15(3):443-8.

23. Chappell S, Daly L, Morgan K, et al. Genetic variants of microsomal epoxide hydrolase and glutamate-cysteine ligase in COPD. The European respiratory journal. 2008;32(4):931-937.

24. Korytina GF, Akhmadishina LZ, Kochetova OV, et al. [Association of cytochrome P450 genes polymorphisms (CYP1A1 and CYP1A2) with the development of chronic obstructive pulmonary disease in Bashkortostan]. Molekuliarnaia biologiia. 2008;42(1):32-41.

25. Zidzik J, Slabá E, Joppa P, et al. Glutathione S-transferase and microsomal epoxide hydrolase gene polymorphisms and risk of chronic obstructive pulmonary disease in Slovak population. Croatian medical journal. 2008;49(2):182-191.

26. Cheng SL, Yu CJ, Yang PC. Genetic polymorphisms of cytochrome p450 and matrix metalloproteinase in chronic obstructive pulmonary disease. Biochemical genetics. 2009;47(7-8):591-601.

27. Faramawy MM, Mohammed TO, Hossaini AM, et al. Genetic polymorphism of GSTT1 and GSTM1 and susceptibility to chronic obstructive pulmonary disease (COPD). Journal of critical care. 2009;24(3):e7-10.

28. Houben JM, Mercken EM, Ketelslegers HB, et al. Telomere shortening in chronic obstructive pulmonary disease. Respir Med. 2009;103(2):230-236.

29. Korytina GF, Akhmadishina LZ, Tselousova OS, et al. [Polymorphism of the genes for antioxidant defense enzymes and their association with the development of chronic obstructive pulmonary disease in the population of Bashkortostan]. Genetika. 2009;45(7):967-976.

30. Mehrotra S, Sharma A, Kumar S, et al. Polymorphism of glutathione S-transferase M1 and T1 gene loci in COPD. International journal of immunogenetics. 2010;37(4):263-267.

31. Penyige A, Poliska S, Csanky E, et al. Analyses of association between PPAR gamma and EPHX1 polymorphisms and susceptibility to COPD in a Hungarian cohort, a case-control study. BMC medical genetics. 2010;11:152.

32. Pietras T, Szemraj J, Witusik A, et al. The sequence polymorphism of MnSOD gene in subjects with respiratory insufficiency in COPD. Medical science monitor : international medical journal of experimental and clinical research. 2010;16(9):Cr427-432.

33. Sørheim IC, DeMeo DL, Washko G, et al. Polymorphisms in the superoxide dismutase-3 gene are associated with emphysema in COPD. Copd. 2010;7(4):262-268.

34. Vibhuti A, Arif E, Mishra A, et al. CYP1A1, CYP1A2 and CYBA gene polymorphisms associated with oxidative stress in COPD. Clinica chimica acta; international journal of clinical chemistry. 2010;411(7-8):474-480.

35. Chen CZ, Wang RH, Lee CH, et al. Polymorphism of microsomal epoxide hydrolase is associated with chronic obstructive pulmonary disease and bronchodilator response. Journal of the Formosan Medical Association = Taiwan yi zhi. 2011;110(11):685-689.

36. Lakhdar R, Denden S, Mouhamed MH, et al. Correlation of EPHX1, GSTP1, GSTM1, and GSTT1 genetic polymorphisms with antioxidative stress markers in chronic obstructive pulmonary disease. Experimental lung research. 2011;37(4):195-204.

37. Lee J, Nordestgaard BG, Dahl M. EPHX1 polymorphisms, COPD and asthma in 47,000 individuals and in meta-analysis. The European respiratory journal. 2011;37(1):18-25.

38. Shukla RK, Kant S, Bhattacharya S, et al. Association of genetic polymorphism of GSTT1, GSTM1 and GSTM3 in COPD patients in a north Indian population. Copd. 2011;8(3):167-172.

39. Thakur H, Gupta L, Sobti RC, et al. Association of GSTM1T1 genes with COPD and prostate cancer in north Indian population. Molecular biology reports. 2011;38(3):1733-1739.

40. Yechshzhanov T, Akparova A, Bersimbay R, et al. Bersimbay. Association of Xenobiotic Detoxification Enzymes Gene Polymorphism in Predisposition of Bronchial Asthma and Chronic Obstructive Pulmonary Disease. Journal of Life Sciences. 2011;5:777-783.

41. Young RP, Hopkins RJ, Hay BA, et al. GSTM1 null genotype in COPD and lung cancer: evidence of a modifier or confounding effect? The application of clinical genetics. 2011;4:137-144.

42. Bose P and Bathri R. Glutathione S-transferase gene polymorphisms (GSTT1, GSTM1, GSTP1) as increased risk factors for asthma and COPD among isocyanate exposed population of Bhopal, India. Research Journal of Recent Sciences. 2012;1:219-223.

43. Putra AC, Tanimoto K, Arifin M, et al. Genetic variations in detoxification enzymes and HIF-1α in Japanese patients with COPD. The clinical respiratory journal. 2013;7(1):7-15.

44. Matokanovic M, Rumora L, Popovic-Grle S, et al. Association of hsp70-2 (+1267A/G), hsp70-hom (+2437T/C), HMOX-1 (number of GT repeats) and TNF-alpha (+489G/A) polymorphisms with COPD in Croatian population. Clin Biochem. 2012;45(10-11):770-774.

45. Begum A, Venkateshwari A, Balakrishna N et al. Association of GSTM1 & HMOX-1 GenePolymorphisms in COPD: A study from South Indian Population.International Journal of Science and Research .2014;3:318-324

46. Dey T, Gogoi K, Unni BG, et al. Role of glutathione S transferase polymorphism in COPD with special reference to peoples living in the vicinity of the open cast coal mine of Assam. PloS one. 2014;9(5):e96739.

47. Taniguchi N, Konno S, Isada A, et al. Association of the CAT-262C>T polymorphism with asthma in smokers and the nonemphysematous phenotype of chronic obstructive pulmonary disease. Annals of allergy, asthma & immunology : official publication of the American College of Allergy, Asthma, & Immunology. 2014;113(1):31-36.e32.

48. Yang L, Li F, Yan M, et al. Association of the CYP1A1 MspI and TNFα-308 polymorphisms with chronic obstructive pulmonary disease in Inner Mongolia. Genetics and molecular research : GMR. 2014;13(2):3209-3217.

49. Zuntar I, Petlevski R, Dodig S, et al. GSTP1, GSTM1 and GSTT1 genetic polymorphisms and total serum GST concentration in stable male COPD. Acta pharmaceutica (Zagreb, Croatia). 2014;64(1):117-129.

50. El Wahsh RA, Essa ES, Bakr RM, et al. GSTM1, GSTT1 and EPHX1 gene polymorphisms and susceptibility to COPD in a sample of Egyptian population. Egyptian Journal of Chest Diseases and Tuberculosis. 2015;64(4):829-836.

51. Gandhi G, Kaur G. Oxidative DNA Damage, Oxidative Stress and Genetic Susceptibility-Prognostic Scores in ‘Missing’ COPD Cases. International Journal of Human Genetics. 2015;15(3):97-119.

52. Stanković M, Nikolić A, Tomović A, et al. Association of Functional Variants of Phase I and II Genes with Chronic Obstructive Pulmonary Disease in a Serbian Population. Journal of medical biochemistry. 2015;34(2):207-214.

53. Khan NA, Kumar N, Husain SA, et al. Association of Xenobiotic Metabolizing Gene Polymorphisms and Chronic Obstructive Pulmonary Disease in Indian Population. Journal of Advanced Research in medicine.2016.3(1):5-14..

54. Akparova A, Abdrakhmanova B, Banerjee N, et al. EPHX1 Y113H polymorphism is associated with increased risk of chronic obstructive pulmonary disease in Kazakhstan population. Mutation research Genetic toxicology and environmental mutagenesis. 2017;816-817:1-6.

55. Cao T, Xu N, Wang Z, et al. Effects of Glutathione S-Transferase Gene Polymorphisms and Antioxidant Capacity per Unit Albumin on the Pathogenesis of Chronic Obstructive Pulmonary Disease. Oxidative medicine and cellular longevity. 2017: 2017: 1-8.

56. Malic Z, Topic A, Francuski D, et al. Oxidative Stress and Genetic Variants of Xenobiotic-Metabolising Enzymes Associated with COPD Development and Severity in Serbian Adults. Copd. 2017;14(1):95-104.

57. Arpaci A, Yalin S, Taskin D, et al. Investigation of antioxidant enzyme polymorphism in chronic obstructive pulmonary disease patients. Biomedical Research (India). 2018;29(9):1906-1913.

58. Ben Anes A, Ben Nasr H, Garrouche A, et al. The Cu/Zn superoxide dismutase +35A/C (rs2234694) variant correlates with altered levels of protein carbonyls and glutathione and associates with severity of COPD in a Tunisian population. Free Radical Research. 2019;53(3):293-303.

59. Du Y, Zhang H, Xu Y, et al. Association among genetic polymorphisms of GSTP1, HO-1, and SOD-3 and chronic obstructive pulmonary disease susceptibility. International journal of chronic obstructive pulmonary disease. 2019;14:2081-2088.

60. Ma X, Wu Y, Zhang L, et al. Comparison and development of machine learning tools for the prediction of chronic obstructive pulmonary disease in the Chinese population. J Transl Med. 2020;18(1):146.

61. Wurtz ET, Brasch-Andersen C, Steffensen R, et al. Heme oxygenase 1 polymorphism, occupational vapor, gas, dust, and fume exposure and chronic obstructive pulmonary disease in a Danish population-based study. Scand J Work Environ Health. 2020;46(1):96-104.

62. Ganbold C, Jamiyansuren J, Tumurbaatar A, et al. The Cumulative Effect of Gene-Gene Interactions Between GSTM1, CHRNA3, CHRNA5 and SOD3 Gene Polymorphisms Combined with Smoking on COPD Risk. International journal of chronic obstructive pulmonary disease. 2021;16:2857-2868.

63. Tacheva T, Zienolddiny-Narui S, Dimov D, et al. The Leucocyte Telomere Length, GSTM1 and GSTT1 Null Genotypes and the Risk of Chronic Obstructive Pulmonary Disease. Curr Issues Mol Biol. 2022;44(8):3757-3769.
