# Supplementary material for: Association between the oxidative stress gene polymorphism and chronic obstructive pulmonary disease risk: a meta-analysis
Source: BMC Pulm Med. 2023 Oct 10;23:384. doi: 10.1186/s12890-023-02625-y (PMC10566167; doi:10.1186/s12890-023-02625-y)
Supplement: Supplementary file 2 — Additional file 2. [file 12890_2023_2625_MOESM2_ESM.docx]

**Identification of studies via other methods**

**Identification of studies via databases and registers**

Records identified from:

Websites (n =0 )

Organisations (n =0 )

Citation searching (n =0 )

etc.

Records removed *before screening*:

Duplicate records removed (n = 15)

Records marked as ineligible by automation tools (n = 10)

Records removed for other reasons (n = 3)

Records identified from*:

Databases (n =126 )

Registers (n =0 )

**Identification**

Records screened

(n =98 )

Records excluded**

(n =22 )

Reports not retrieved

(n =0 )

Reports sought for retrieval

(n = 0)

Reports sought for retrieval

(n =76 )

Reports not retrieved

(n =10 )

**Screening**

Reports assessed for eligibility

(n = 0)

Reports excluded:

Reason 1 (n =0 )

Reason 2 (n = 0)

Reason 3 (n = 0)

etc.

Reports assessed for eligibility

(n = 66)

Reports excluded:

Reason 1 (n =3 )

Reason 2 (n =0 )

Reason 3 (n =0 )

etc.

Studies included in review

(n =63 )

Reports of included studies

(n =63 )

**Included**

*Consider, if feasible to do so, reporting the number of records identified from each database or register searched (rather than the total number across all databases/registers).

**If automation tools were used, indicate how many records were excluded by a human and how many were excluded by automation tools.

*From:*  Page MJ, McKenzie JE, Bossuyt PM, Boutron I, Hoffmann TC, Mulrow CD, et al. The PRISMA 2020 statement: an updated guideline for reporting systematic reviews. BMJ 2021;372:n71. doi: 10.1136/bmj.n71. For more information, visit: <http://www.prisma-statement.org/>
